# Introducing gene deletions by mouse zygote electroporation of Cas12a/Cpf1

**DOI:** 10.1007/s11248-019-00168-9

**Published:** 2019-09-03

**Authors:** Charles-Etienne Dumeau, Asun Monfort, Lucas Kissling, Daan C. Swarts, Martin Jinek, Anton Wutz

**Affiliations:** 1 Department of Biology, Institute of Molecular Health Sciences, Swiss Federal Institute of Technology, Zurich, Switzerland; 2 Department of Biochemistry, University of Zurich, Zurich, Switzerland; 3 Present Address: Laboratory of Biochemistry, Department of Agrotechnology and Food Sciences, Wageningen University, 6708 WE Wageningen, The Netherlands

**Keywords:** CRISPR-Cas, Cas12a, Cpf1, Electroporation, Mutation, Mouse embryo, Gene deletion

## Abstract

CRISPR-associated (Cas) nucleases are established tools for engineering of animal genomes. These programmable RNA-guided nucleases have been introduced into zygotes using expression vectors, mRNA, or directly as ribonucleoprotein (RNP) complexes by different delivery methods. Whereas microinjection techniques are well established, more recently developed electroporation methods simplify RNP delivery but can provide less consistent efficiency. Previously, we have designed Cas12a-crRNA pairs to introduce large genomic deletions in the *Ubn1*, *Ubn2*, and *Rbm12* genes in mouse embryonic stem cells (ESC). Here, we have optimized the conditions for electroporation of the same Cas12a RNP pairs into mouse zygotes. Using our protocol, large genomic deletions can be generated efficiently by electroporation of zygotes with or without an intact zona pellucida. Electroporation of as few as ten zygotes is sufficient to obtain a gene deletion in mice suggesting potential applicability of this method for species with limited availability of zygotes.

**Electronic supplementary material:**

The online version of this article (10.1007/s11248-019-00168-9) contains supplementary material, which is available to authorized users.

## Introduction

Clustered regularly interspaced short palindromic repeat (CRISPR) associated nucleases are components of bacterial and archaeal adaptive defense mechanisms against invasive nucleic acids such as plasmids or viral DNA (Horvath and Barrangou 2010). These nuclease enzymes associate with short CRISPR RNAs (crRNA) that guide them to complementary target sequences in the genome. As the CRISPR associated nucleases Cas9 and Cas12a can be programmed with crRNA sequences of choice, they have been repurposed into a versatile genome editing tool for many biological tissues in different species (Hai et al. 2014; Hwang et al. 2013; Jinek et al. 2012; Lee et al. 2019; Mali et al. 2013; Sung et al. 2014; Wang et al. 2013). During genome editing, both Cas9 and Cas12a generate DNA double strand breaks (DSB) at sequences that are complementary to the crRNA sequence (Dong et al. 2016; Fonfara et al. 2016; Jinek et al. 2012; Zetsche et al. 2015), which subsequently trigger cellular DNA repair processes. Two distinct cellular DNA repair mechanisms are known: Homology directed repair (HDR) requires a template and can be used to engineer precise genomic mutations, whereas non-homologous end joining (NHEJ) generates small insertions or deletions (indels) at the site of the break (Cong et al. 2013; Jinek et al. 2013; Mali et al. 2013). Cas9 has been exploited in combination with both repair mechanisms for developing highly efficient methods for producing genomic mutations in mice (Hai et al. 2014; Sung et al. 2014; Wang et al. 2013). Using Cas9 nucleases in combination with targeting vectors has further facilitated gene targeting in mouse zygotes by pronuclear microinjection (Wang et al. 2015). Recently, it could be demonstrated that microinjection of Cas9-crRNA RNP complexes can increase the efficiency of gene targeting in mouse zygotes (Jung et al. 2017). These studies suggest that ESC technology might be replaced by direct engineering of the zygotic genome for gene targeting.

Nevertheless, microinjection techniques take considerable time to perform and require extensive training for obtaining high rates of transgenesis and embryo survival. To overcome these difficulties, square wave electroporation techniques have been developed to introduce Cas9 mRNA/crRNA or Cas9-RNP complexes into mouse and rat zygotes using different electroporation systems (Hashimoto et al. 2016; Kaneko and Mashimo 2015; Kaneko et al. 2014; Qin et al. 2015; Troder et al. 2018). Zygote electroporation requires establishing suitable parameters that facilitate uptake of Cas9 but also limit damage to the embryo. A first poring pulse series is applied to permeabilize the zona pellucida and the cell membrane of the zygote. Subsequently, a second transfer pulse series delivers the Cas9 mRNA/crRNA or the Cas9-RNP complex into the cytoplasm (Hashimoto and Takemoto 2015; Hashimoto et al. 2016). Further refinement of zygote electroporation has focused on poring pulse parameters for optimizing RNP delivery and zygote survival (Modzelewski et al. 2018). Although zygote electroporation requires skills for embryo handling, microinjection skills are dispensable, making this technique accessible to a broad range of scientists.

More recently, also Cas12a-RNP complexes have been used for generating indel mutations by electroporation into zygotes (Hur et al. 2016). Cas12a also generates DSB but has divergent characteristics compared to Cas9 including differences in crRNA binding, DNA recognition, and DNA cleavage mechanism, which expands the genome editing tool box (Swarts and Jinek 2018). Cas12a nucleases are guided to specific DNA sequences by crRNAs which are 40–44 nucleotides long and can easily be obtained by chemical synthesis. In contrast, Cas9 nucleases are guided by longer sgRNAs (~ 101 nucleotides) or by dual-RNA consisting of a separate tracrRNA (~ 75 nucleotides) and crRNA (39–42 nucleotides) (Kaneko and Mashimo 2015; Modzelewski et al. 2018; Swarts and Jinek 2018). The use of chemically synthesized crRNAs, which can be obtained at high purity, guaranteed length, and exact sequence, make experimental planning easier and provide reagents of a standardized quality for experiments involving animals. Shorter RNAs can be more cost effective to synthesize suggesting that Cas12a nucleases might have advantages over Cas9 nucleases for genetic engineering of ESCs or zygotes by RNP electroporation. While the molecular mechanisms of Cas12a nucleases have been extensively characterized (Dong et al. 2016; Fonfara et al. 2016; Zetsche et al. 2015), in vivo applications of Cas12a remain less well established compared to in vivo applications of Cas9. In a previous study, we have provided a protocol for generating large deletions in mouse ESCs by electroporation of Cas12a RNP complexes (Kissling et al. 2019). For this, two CRISPR-Cas nucleases are designed that cleave the genome at separated sites within a gene locus such that loss of the intervening sequence will result in a deletion. Large genomic deletions are advantageous for mutation of genes for which critical domains are unknown or for which specific antisera are not available. Removal of a large segment of the gene locus increases the probability for generating a strong and likely complete loss-of-function mutation. Small deletions including frame shift mutations afford less certainty about residual gene function, especially when alternative splicing needs to be considered. In our previous study, we identified pairs of crRNAs for efficient engineering of Cas12a-RNP complex-mediated 10 kilobasepair (kb), 17 kb, and 13 kb long genomic deletions within the *Ubinuclein 1* (*Ubn1*), *Ubinuclein 2* (*Ubn2*), and *RNA binding motif protein 12* (*Rbm12*) genes, respectively.

Here, we extend our earlier study in mouse ESCs to engineer large genomic deletions in mouse zygotes through electroporation of Cas12a-RNP complexes.

## Materials and methods

### Zygote production and partial zona removal

For obtaining zygotes, 6–8-week-old Crl:CD1(ICR) females were superovulated and mated to Crl:CD1(ICR) males the night before zygote collection. Pregnant Mare Serum Gonadotropin (PMSG, Avivasysbio cat. no. OPPA1037 5000 IU) and human Chorionic Gonadotropin (hCG, MSD animal health Chorulon 5000, Swissmedic cat. no. 49451) were diluted in PBS at 50 IU/ml and stored at − 20 °C in 1 ml aliquots. 5 IU PMSG, and hCG were administered per mouse by intraperitoneal injection using 27G hypodermic needles 66 and 19 h before collection, respectively. Zygotes were isolated from the ampulla of dissected oviducts in M2 medium (Sigma, cat. no. M7167). Cumulus masses were treated with hyaluronidase (Sigma cat. no. H4272) at 0.5 mg/ml in M2 medium, washed 3 times in M2 medium, and incubated at 37 °C, 5% CO_2_ in 60 mm center well culture dishes (Corning Incorporated, Costar cat. no. 3260) in KSOM medium (Millipore Embryomax cat. no. MR-020P-5F) supplemented with 1% NEAA (NEAA 100X Gibco, cat. no. 11140035) before electroporation. Zygotes with abnormal morphology were discarded after visual inspection. For partial removal of the zona pellucida zygotes were treated with acidic Tyrode’s solution (Sigma, cat. no. T1788) for 20–30 s under a stereomicroscope to follow the thinning of the zona, washed 3 times in M2 medium and used for electroporation (AT group). Glass pipettes for handling zygotes and embryos were hand drawn over a flame from Hirschmann 100 µl micropipettes (Sigma cat. no. Z61163). All animal experiments were carried out in compliance with cantonal and federal animal welfare regulations.

### Cas12a nuclease RNP assembly

Production of the Cas12a-NLS-NLS-eGFP-NLS fusion protein (Cas12a nuclease), design of crRNAs, verification of DNA cleavage activity in vitro and PCR analysis of genomic deletions in *Ubn1*, *Ubn2*, and *Rbm12* were previously described (Kissling et al. 2019). crRNA sequences are listed (Suppl. Table 2). The Cas12a nuclease protein was stored as a 12 µM stock solution in SEC buffer (20 mM HEPES pH 7.5, 500 mM KCl, 1 mM DTT) at − 80 °C. For engineering deletions, pairs of Cas12a RNPs were used for electroporation. The two RNP complexes were assembled separately. For RNP complex assembly (15 µl total volume) 8.3 μl Cas12a protein (12 μM) in SEC buffer, 1.5 μl crRNA (100 μM) in ddH_2_O, and 5.2 μl of Cas12a buffer (Tris-HCl 8 mM pH7.4, EDTA 0.1 mM, MgCl_2_ 2 mM) were mixed in an Eppendorf tube. The final concentrations for Cas12a protein, and crRNA were 6.64 µM, and 10 µM, respectively. Assembly was carried out by incubation for 10 min at room temperature. Thereafter RNP complexes were stored at 4 °C until use for electroporation. RNP complexes that were stored over night maintained activity. The two independently assembled RNP complexes were mixed in a 1:1 ratio (30 µl final volume) before electroporation and kept on ice.

### Zygote electroporation

Electroporations were carried out with a NEPA21 type II electroporator (Nepagene), a 5 µl electrode chamber with a gap width of 1 mm (Nepagene CUY501P1-1.5), and connection cables (Nepagene C115CB, and C117). The instrument was operated following the instructions of the manufacturer and used with the settings listed in Table 1. The electrode chamber was filled with 5 µl of Cas12a nuclease pair mix. Zygotes (up to 40) were washed through 3 micro-drops of RNP mix and transferred into the electrode chamber by mouth pipetting. The sample impedance was measured on the NEPA21 instrument and adjusted to a value within the range from 190 and 200 Ohm by adding or removing RNP mix. After electroporation zygotes were transferred into center well culture dishes with KSOM medium supplemented with 1% NEAA.

**Table 1:** Settings of the NEPA21 electroporation system for zygote electroporation using a 5 µl electrode chamber

	Voltage (V)	Length (ms)	Interval (ms)	Number of pulses	D. Rate (%)	Polarity
Poring pulse	30	3	100	6	10	+
Transfer pulse	5	50	50	5	40	+ / −

### Derivation of ESC lines

Mouse embryos were cultured in KSOM medium supplemented with 1% NEAA to the 8-cell stage and then transferred into KSOM media (Millipore Embryomax cat. no. MR-020P-5F) supplemented with 1% NEAA, 2% Essential amino-acid (EAA 50X Gibco, cat. no.11130051), 1 mg/ml glucose, 1 µM PDO325901, and 3 µM CHIR99021 until they reached the late blastocyst stage. Inner cell masses were isolated by immunosurgery (Solter and Knowles 1975), and cultured in N2B27+2i/LIF medium for feeder free ESC derivation as previously published (Nichols et al. 2009a, b).

### Genotyping by PCR

ESC lines were genotyped by PCR reactions using primer pairs spanning the deletion, and flanking the cleavage sites as previously published (Kissling et al. 2019). A PCR master mix was prepared on ice for the number of samples tested. One PCR reaction is composed of 16.1 µl ddH2O, 2 µl Thermopol Buffer (NEB), 0.4 µl 10 mM dNTP mix, 0.4 µl 10 µM forward primer, 0.4 µl 10 µM reverse primer, 0.1 µl TAQ polymerase (NEB M0267 at 5000U/ml) and 0.6 µl DNA template. Thermocycler programs were setup following the guidelines of the supplier (NEB). Primer sequences and pairs are provided in Suppl. Tables 3 and 4 with corresponding annealing temperatures.

## Results

**Fig. 1:**
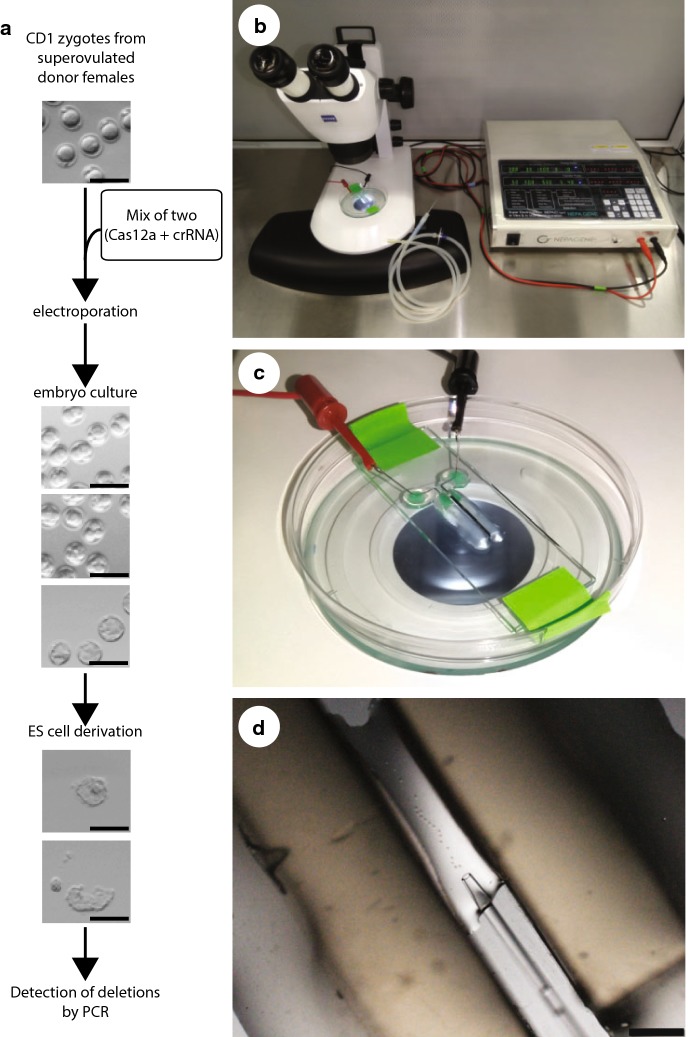
Experimental setup and overview of the procedure for zygote electroporation. **a** Scheme of the experimental strategy for engineering gene deletions by zygote electroporation. Scale bar, 200 µm. **b** Setup of the work area for zygote electroporation and placement of a NEPA21 electroporation system and stereomicroscope. **c** Picture of the electroporation chamber used for electroporation in 5 µl volume and electrical connections. **d** Placement of mouse zygotes between the electrodes just prior to electroporation using a handling pipette under the stereomicroscope. Scale bar, 1 mm

The NEPA electroporation system has been previously used for zygote electroporation (Hashimoto and Takemoto 2015; Hashimoto et al. 2016; Hur et al. 2016) and is available with electroporation chambers that are specifically designed for use with embryos. We adopted a workflow for electroporation of CD1 zygotes with an equimolar mix of two Cas12a RNPs for inducing genomic deletions within genes. After electroporation we cultured embryos in vitro to the blastocyst stage to derive ESC lines that represent clonal populations where genotyping of the genetic modifications can be performed by PCR (Fig. 1a). The setup for electroporation requires a mouth pipette, a stereomicroscope for embryo handling, and the NEPA21 electroporator (Fig. 1b). The NEPA electroporation system enables measurements of sample conductance and a precise reading of electrical intensity and energy delivered, which improves the reproducibility of the experimental conditions. Previous studies have reported electroporation into zygotes in a volume of 100 µl (Hur et al. 2016; Kaneko and Mashimo 2015; Kaneko et al. 2014) using an electrode chambers with an electrode gap of 5 mm. More recently a 5 µl chamber with an electrode gap of 1 mm has become available reducing the reagent volume. An earlier study has successfully used such a chamber for electroporation of Cas9 nucleases into mouse zygotes (Teixeira et al. 2018). We decided to test this electroporation chamber for electroporation of Cas12a nucleases (Fig. 1c, d). Initially, we evaluated the effect of poring pulse voltage with a constant poring pulse number of 4 on embryo survival (Suppl. Fig. 1, and Suppl. Table 1). With a poring pulse of 30 V embryo survival approached 100%, while 40 and 50 V poring pulses gave also high embryo survival rates (on average 90% and 84%, respectively). However, we did not detect successful Cas12a-RNP complex-mediated deletions with these series of 4 pulses. We therefore increased the number of pulses and the interval between pulses. Trials with adjusted poring pulse voltage of 45 V over 1 mm electrode gap width, which corresponds to the same electrical field strength as the original conditions using 225 V over a 5 mm electrode gap, resulted in substantial lysis of zygotes and caused loss of embryos (data not shown). We did not further pursue experiments with higher voltages and selected 30 V as the highest voltage that was compatible with zygote survival in our preliminary experiments. Using 6 poring pulses of 30 V, 3 ms length (Modzelewski et al. 2018), 100 ms intervals, 10% decay rate, and positive (+) polarity followed by 5 transfer pulses of 5 V, 50 ms length, 50 ms interval, 40% decay rate, and alternating (+/−) polarity we successfully detected Cas12a-RNP complex-mediated deletion with little or no loss of embryos (data not shown).

To assess the reproducibility of small volume zygote electroporation, we aimed at generating deletions in three genes, *Ubn1*, *Ubn2*, and *Rmb12*. For these genes no mutations have been described in mice to date, and we have identified efficient Cas12a-crRNA combinations in mouse ESC electroporation experiments in a previous study (Kissling et al. 2019). Although a large number of Cas12a-crRNA combinations showed excellent cleavage of their DNA targets in vitro, only a subset of Cas12a-crRNA RNPs yielded efficient deletions in ESCs. This discrepancy was not trivially explained by sequence context or local chromatin accessibility as nucleases targeting opposite strands in close proximity showed substantial differences as evidenced by assessing NHEJ mutations (Kissling et al. 2019). This suggests that combinations of the accessibility, sequence preference, and repair processes might influence the effective rates in cellular systems with which deletions can be obtained. Empirical determination of cleavage and deletion efficiency is therefore a prerequisite for testing the methodology for zygote genetic engineering.

We used the same Cas12a-crRNA RNPs for small volume zygote electroporation in this study. A total of 251 zygotes were collected for electroporation. For each experiment we divided the zygotes into two groups. The first group of zygotes was treated with acidic Tyrod’s solution (AT) for 20–30 s to partially remove the zona pellucida. The second group of zygotes was used for electroporation with an intact zona pellucida (NoAT). Thirty minutes after electroporation we monitored embryo lysis by inspection under the microscope. The overall survival rates were 85%, and 92% for the NoAT and AT groups, respectively (Table 2). Subsequently, zygotes were cultured in vitro and their development was followed through inspection under a stereomicroscope. For the NoAT and AT groups 98% and 95% of the embryos reached the 2 cell-stage, 68% and 62% developed into morulae, and 37% and 36% to blastocysts, respectively (Table 2). To obtain clonal cell populations for analysis of genetic modifications, we subsequently derived 30 ESC lines from blastocysts for each group with a derivation rate of 28% and 26% for the NoAT and AT group, respectively (Table 2). Since ESC lines are derived from individual epiblast cells the genotype can be directly assessed. In contrast, analysis of embryos can be more difficult as cells of heterogeneous allelic constitution can result from Cas12a nuclease induced cleavage after DNA replication or in cleavage stage embryos. Analysis of resulting chimeric embryos is complicated by the existence of cells with potentially divergent genotypes. This problem is avoided by analyzing clonal ESC populations.

**Table 2:** Survival and in vitro development of mouse embryos after electroporation of zygotes with either intact or partially removed zona pellucida with acidic Tyrode's solution (AT)

Gene	Zygotes	AT	Survival^a^	2-Cell^b^	Morula^b^	Blastocyst^b^	ESC lines^b^	Deletion^c^	Inversion^c^
*Ubn1*	47	No	47 100%	45 96%	23 49%	10 21%	10 21%	6 60%	1 10%
*Ubn2*	39	No	28 72%	28 100%	27 96%	13 46%	7 25%	3 43%	0 0%
*Rbm12*	40	No	32 80%	32 100%	23 72%	17 53%	13 41%	3 23%	3 23%
Total	126	No	107 85%	105 98%	73 68%	40 37%	30 28%	12 40%	4 13%
*Ubn1*	50	Yes	50 100%	45 90%	23 46%	13 26%	13 26%	7 54%	1 8%
*Ubn2*	38	Yes	32 84%	32 100%	30 94%	13 41%	11 34%	7 64%	0 0%
*Rbm12*	37	Yes	33 89%	32 97%	18 55%	15 45%	6 18%	1 17%	1 17%
*Total*	125	Yes	115 92%	109 95%	71 62%	41 36%	30 26%	15 50%	2 7%

^a^Percentages are based on the number of zygotes that were used for electroporation

^b^Percentages are based on the number of zygotes that survived electroporation

^c^Percentages are based on the number of ES cell lines that were derived

**Fig. 2:**
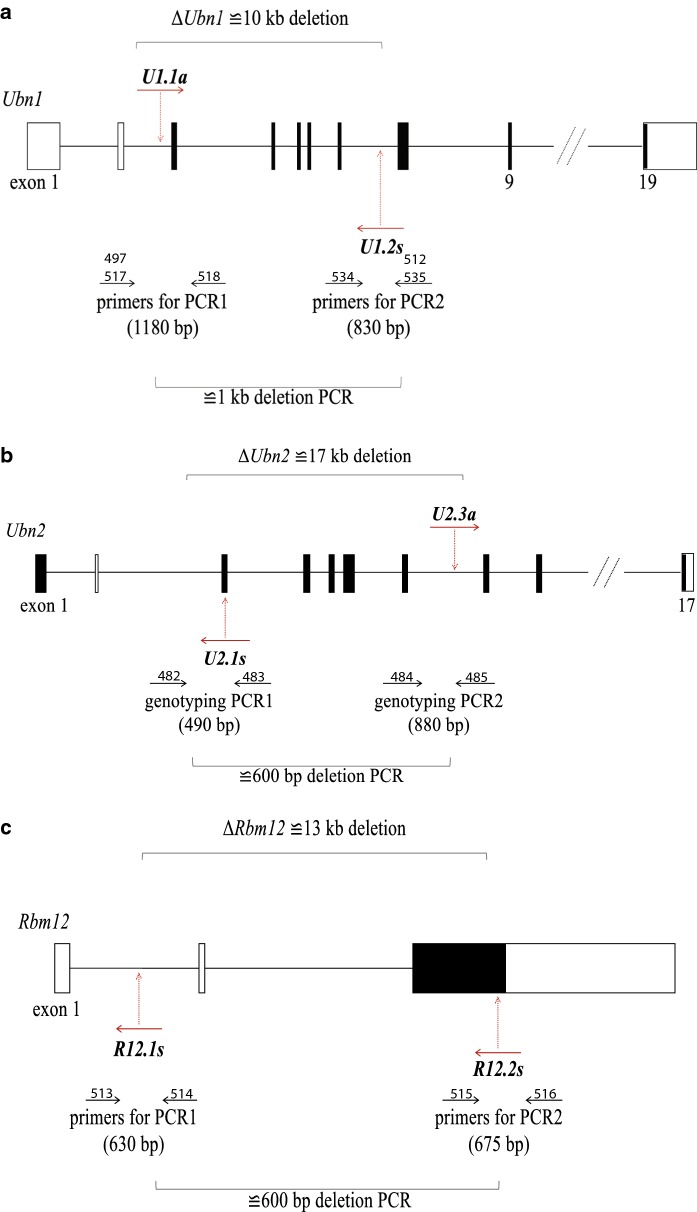
Schematic representation of the strategies for engineering deletions in the *Ubn1*, *Ubn2*, and *Rbm12* genes. The structure of the gene locus is shown along with the locations of crRNAs (red full arrows), predicted cleavage sites (dashed red arrows), and PCR primers (black arrows) for **a***Ubn1*, **b***Ubn2*, and **c***Rbm12*. (Color figure online)

**Fig. 3:**
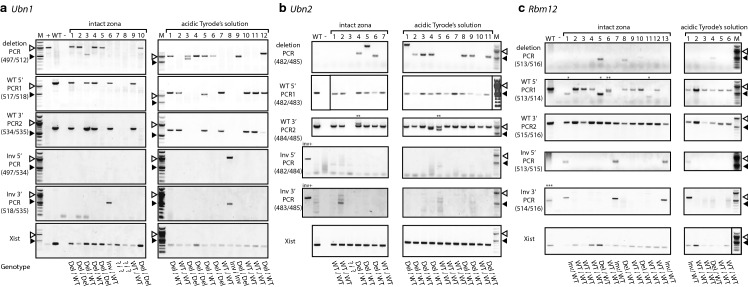
Analysis of gene deletions in ESCs established after zygote electroporation. Representative images of agarose gel electrophoresis of PCR products using primers specific for deletions in the **a***Ubn1*, **b***Ubn2*, and **c***Rbm12* genes. Lanes with positive control sample (+), wild type control (WT), no template control (−), inversion positive control (inv+), molecular weight marker (M), and samples from ESC lines derived from electroporated embryos (numerical lane labels) are shown. No inversion positive control are available for *Ubn1* and *Rbm12*. The positions of the 500 bp (black arrow head) and 1 kb (white arrow head) size marker fragments are indicated. Primer pairs used are indicated for each gel. The genotype is given for each cell line (below); alleles are categorized for deletion (Del), inversion (Inv), wild type (WT) and uncharactized event (?). Annotation: (*) potential complex rearrangement, (**) potential complex rearrangement or polyclonal ESC line, (***) potential unspecific fragment detected in samples

The presence or absence of genomic deletions was detected by PCR using primer pairs that flank the genomic positions of the Cas12a cleavage sites (Fig. 2a–c). We tested the samples as well for the presence of wild-type sequence spanning both cleavage sites (Fig. 2a–c referred to as PCR1 and PCR2 for the in 5′ and 3′ flanking region, respectively). Finally we looked for sequence inversion by inverting the primer pair combination of PCR1 and PCR2. We observed frequencies of gene deletions ranging from 17 to 64% with an overall frequency of deletion of 40% and 50% for the NoAT an AT group, respectively (Fig. 3a–c and Table 2). There was a considerable variation in the deletion frequency between the genes. The lowest frequency of deletions was observed for the *Rbm12* gene with 23% and 17% of ESC lines carrying deletions for the NoAT and AT group, respectively. The highest deletion frequencies were observed for *Ubn1* in the NoAT group with 60%, and for *Ubn2* in the AT group with 64% of ESC lines carrying deletions. We further characterized several deletions by sequencing of PCR products (Suppl. Fig. 2). We also detected sequence inversion for *Ubn1* and *Rbm12* with an overall frequency of 13% and 7% for the NoAT and AT group, respectively. Taken together deletions and inversions occurred with an average frequency of 53% and 57% for the NoAT and AT group, respectively. We did not detect a statistically significant difference between the NoAT and AT groups suggesting that treatment of zygotes with acidic Tyrode's solution does neither increase nor decrease the efficiency of zygote electroporation under our experimental conditions (Fig. 3 and Table 2).

## Discussion

In our study we developed a method for small volume zygote electroporation of Cas12a-crRNA RNPs and demonstrate its use for generating large gene deletions. Outbred CD1 embryos were used for zygote production in this study. However, further experiments in the inbred C57BL6/J background showed that founder mice with a homozygous *Ubn1* deletion were generated using the same conditions (Suppl. Fig. 3). This observation demonstrates that the efficiency of the procedure is not dependent on the genetic background of the zygotes. The ability for generating large deletions is desirable for the analysis of genes that have not been extensively characterized and for which critical sequences cannot be predicted with reasonable certainty. Removal of a large part of the coding exons or gene regulatory regions ensures that a strong and potentially complete loss of function mutation will be obtained. Nevertheless, generation of large deletions might delete regulatory elements that are not associated with the gene of interest and effects on other genes might influence the resulting phenotype. Often specific antisera are unavailable for genes that have not been studied and, hence, independent confirmation of the absence of a protein product is not an experimental possibility. Generating deletions is thought to be considerably more difficult than obtaining indel mutations as it requires the simultaneous cleavage at two sites and a fortuitous DNA repair event that joins the flanking sequences. We obtained a small number of ESC lines that neither yielded expected PCR products for a deletion, an inversion, nor a wildtype allele (Fig. 3, samples marked with genotype ?/?). This observation is likely explained by larger rearrangements of the gene locus that has been previously reported to accompany CRISPR-mediated genetic engineering (Boroviak et al. 2017; Kosicki et al. 2018; Shin et al. 2017) and can include unpredicted larger deletions (Owens et al. 2019) leading to a loss of primer binding sites. Moreover, we observed two clones that showed two wildtype 3′ PCR products, one that showed two wildtype 5′ PCR products, and three clones that showed lower weight wildtype 5′ PCR products (Fig. 3; lanes marked with *, ** and ***), which could be results of smaller rearrangements. However, in these cases we cannot fully rule out the possibility of polyclonal ESC lines. Using a method with small electroporation volumes and two Cas12a-crRNA RNPs at high concentration, we were able to generate 10, 17, and 13 kb long deletions in *Ubn1*, *Ubn2*, and *Rbm12*, respectively. The overall frequency of deletions that was observed in our experiments was 56%, 55%, and 21% for *Ubn1*, *Ubn2*, and *Rbm12,* respectively. This observation suggests that the high concentration of Cas12a RNP nuclease pairs used in our experiments are favorable for generating deletions.

**Fig. 4:**
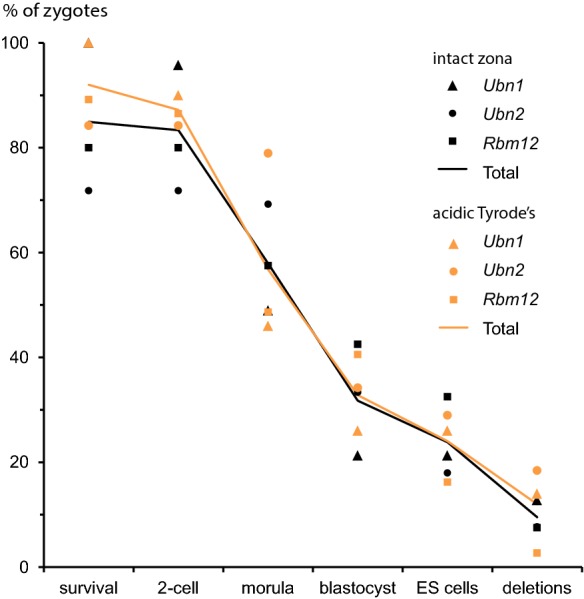
Efficiency of gene deletions by electroporation of Cas12a RNP complexes into zygotes. Embryo development and gene deletions were determined for zygotes that were used for electroporation either with intact zona pellucida (black lines and markers) or whose zona had been partially removed by treatment with acidic Tyrode's solution (orange line and markers). Percentages are calculated relative to the number of zygotes used for the experiment and plotted for each gene (markers) and combined for all 3 genes (lines). (Color figure online)

Cas12a can be a relevant alternative to Cas9 for engineering the mouse genome. In our experiments the use of shorter synthetic Cas12a crRNA guides is cost effective and simplifies project setup by using reagents of a standardized quality. In our experience the vast majority of assembled Cas12a RNP nucleases showed specific nuclease activity in in vitro cleavage reactions using plasmids or PCR products as templates (Kissling et al. 2019). The ability to test the activity of Cas12a RNPs in vitro is important for ensuring that experiments that involve laboratory animals are carried out under optimal conditions. Recently, Cas12a protein has become available from commercial suppliers facilitating the use of Cas12a RNP complexes in a wider range of laboratories. In our study we present an efficient method that (1) minimizes reagents by electroporation in a small volume, (2) takes advantage of the synthetic short Cas12a crRNAs, and (3) avoids partial zona removal for improving consistency and minimizing embryo loss. From our data we estimate that at least one gene deletion can be expected when using as few as 10 zygotes (Fig. 4). The high efficiency and rapid setup suggest potential of electroporation for engineering deletions in species for which limited numbers of zygotes are available or for large scale genomics initiatives that aim at systematic screening a larger number of genes.

## Electronic supplementary material

Below is the link to the electronic supplementary material.

Supplementary file1 (PDF 340 kb)
