# Supplementary material for: Introducing gene deletions by mouse zygote electroporation of Cas12a/Cpf1
Source: Transgenic Res. 2019 Sep 3;28(5):525–35. doi: 10.1007/s11248-019-00168-9 (PMC6848045; doi:10.1007/s11248-019-00168-9)

Electronic Supplementary Material

Supplementary Tables

Suppl. Table 1 Development to 2-cell stage of *in vitro* cultured zygotes after electroporation using 4 poring pulse series of 30, 40 and 50 volts.

Voltage (V)	Length (ms)	Interval (ms)	Number of pulses	Impedance (kΩ)	Zygotes	2-cell (%)
30	3.5	50	4	0.200	25	25 (100%)
30	3.5	50	4	0.217	20	20 (100%)
30	3.5	50	4	0.228	25	25 (110%)
40	3.5	50	4	0.211	33	31 (94%)
40	3.5	50	4	0.204	25	24 (96%)
40	3.5	50	4	0.221	20	19 (95%)
40	3.5	50	4	0.238	23	23 (100%)
40	3.5	50	4	0.220	11	9 (82%)
40	3.5	50	4	0.211	11	5 (45%)
50	2.5	50	4	0.203	6	5 (83%)
50	2.5	50	4	0.196	26	26 (100%)
50	3.5	50	4	0.207	19	18 (95%)
50	3.5	50	4	0.217	25	18 (72%)
50	3.5	50	4	0.205	11	6 (55%)

Suppl. Table 2 Sequences of crRNAs used for this study

Gene	crRNA pair	PAM/target sequence
Ubn 1	U1.1a	TTTGguuugaccuggcccuucuuu
	U1.2s	TTTAccucaguugccugcuuguca
Ubn 2	U2.1s	TTTAcgggcuuucccacccugcaaa
	U2.3a	TTTGcaggcugcugagaauauaguu
Rbm12	R12.1s	TTTAccugacaacaaucagggcaa
	R12.2s	TTTAagucaaugacagcagcugug

Suppl. Table 3 Sequences of PCR primers used for this study

Gene	PCR primers	#	Sequence (5'–3')
Rbm12	R12PCR1 Fw	***516***	GATGGTTCCAGGATCTGAAGTGTG
Rbm12	R12PCR1 Rv	***515***	GTTGGTCTGGACTACAACAGAGAGTG
Rbm12	R12PCR2 Fw	***514***	CGGTTGGAAATAGTGGTTTGCCTG
Rbm12	R12PCR2 Rv	***513***	GTAAAACAGCCAACCAATGCTCTGTG
Ubn1	U1PCR1 Fw	***497***	ACTGACTGGGGGATTTCTTC
Ubn1	U1PCR2 Rv	***512***	TGATGTTCTGCACTGTCCTG
Ubn1	U1PCR1 fw	***517***	ACAACAAACCCCAAGTTTGGA
Ubn1	U1PCR1 Rv	***518***	GGTCCATGTGAACACTGGTAGC
Ubn1	U1PCR2 Fw	***534***	ACTCTCTGACGGATTTGGAC
Ubn1	U1PCR2 Rv	***535***	CCATGCTAAAGACRGCTTGG
Ubn2	U2PCR1 Fw	***482***	GCAAGGTATGGGGAGATCCAG
Ubn2	U2PCR1 Rv	***483***	CACCATATGGCCCACAGTCG
Ubn2	U2PCR2 Fw	***484***	GAGTCTGTGCTGTTAAAGCCACTG
Ubn2	U2PCR2 Rv	***485***	ACATGTATGCAGATGTGGGTGAATGC

Suppl. Table 4 List of PCR primer pairs with corresponding annealing temperature (Tm) used for genotyping

Gene	Target	Primer pair	Tm (°C)
Ubn1	Deletion	497/512	55
	WT 5’	517/518	60
	WT 3’	534/535	55
	Inv 5’	497/534	55
	Inv 3’	518/535	55
Ubn2	Deletion	482/485	60
	WT 5’	482/483	58
	WT 3’	484/485	60
	Inv 5’	482/484	58
	Inv 3’	483/485	58
Rbm12	Deletion	513/516	62
	WT 5’	513/514	62
	WT 3’	515/516	58
	Inv 5’	513/515	62
	Inv 3’	514/516	58

Supplementary Figures

Suppl. Fig. 1 Development of zygotes to the 2-cell stage after electroporation using 30, 40, and 50 V poring pulse series. Average data from Suppl. Table 1 is plotted and the number of zygotes (n) is indicated.

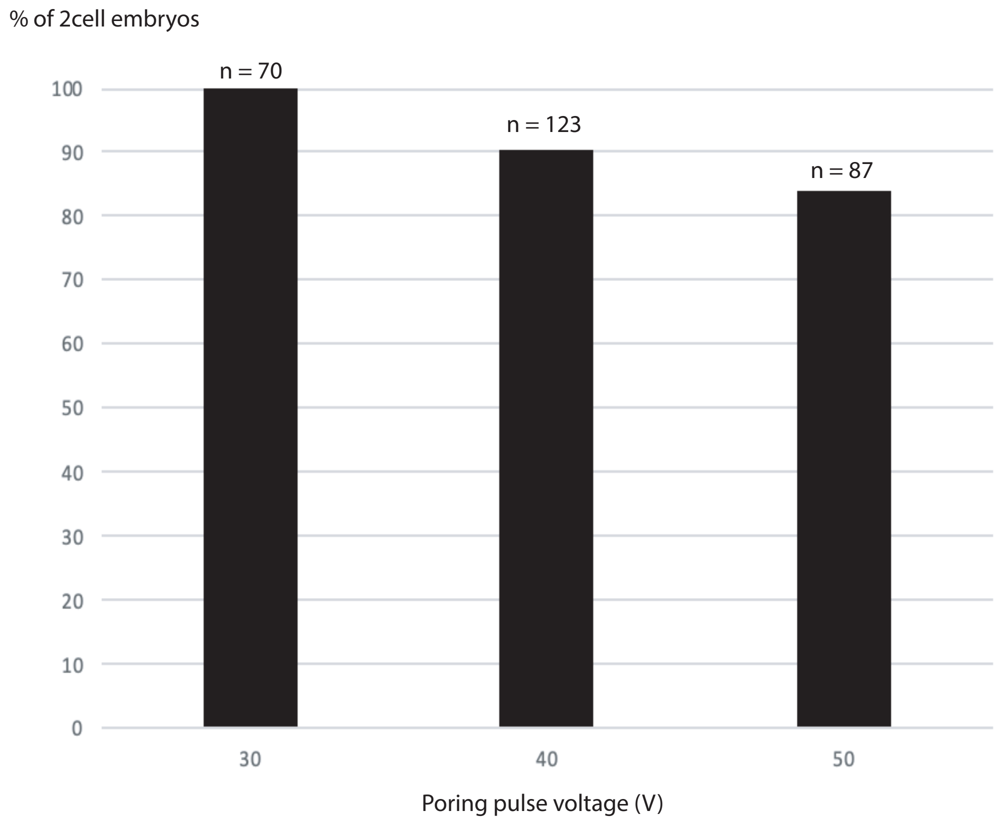

Suppl. Fig. 2 Alignment of DNA sequences obtained from PCR products from ESC lines using deletion specific primers for **a** *Ubn1*, **b** *Ubn2* and **c** *Rbm12* (see Fig. 2a-c). Black line; wild type DNA template for alignment, dashed black line; skipped sequence in the DNA template, red line; aligned sequences of the PCR product, white line; sequence that is not aligned, orange arrow; crRNA used for creating the deletion and dark orange; PAM sequence. ESC lines are annotated for treatment with acidic Tyrod's solution (AT) or no treatment (NoAT).

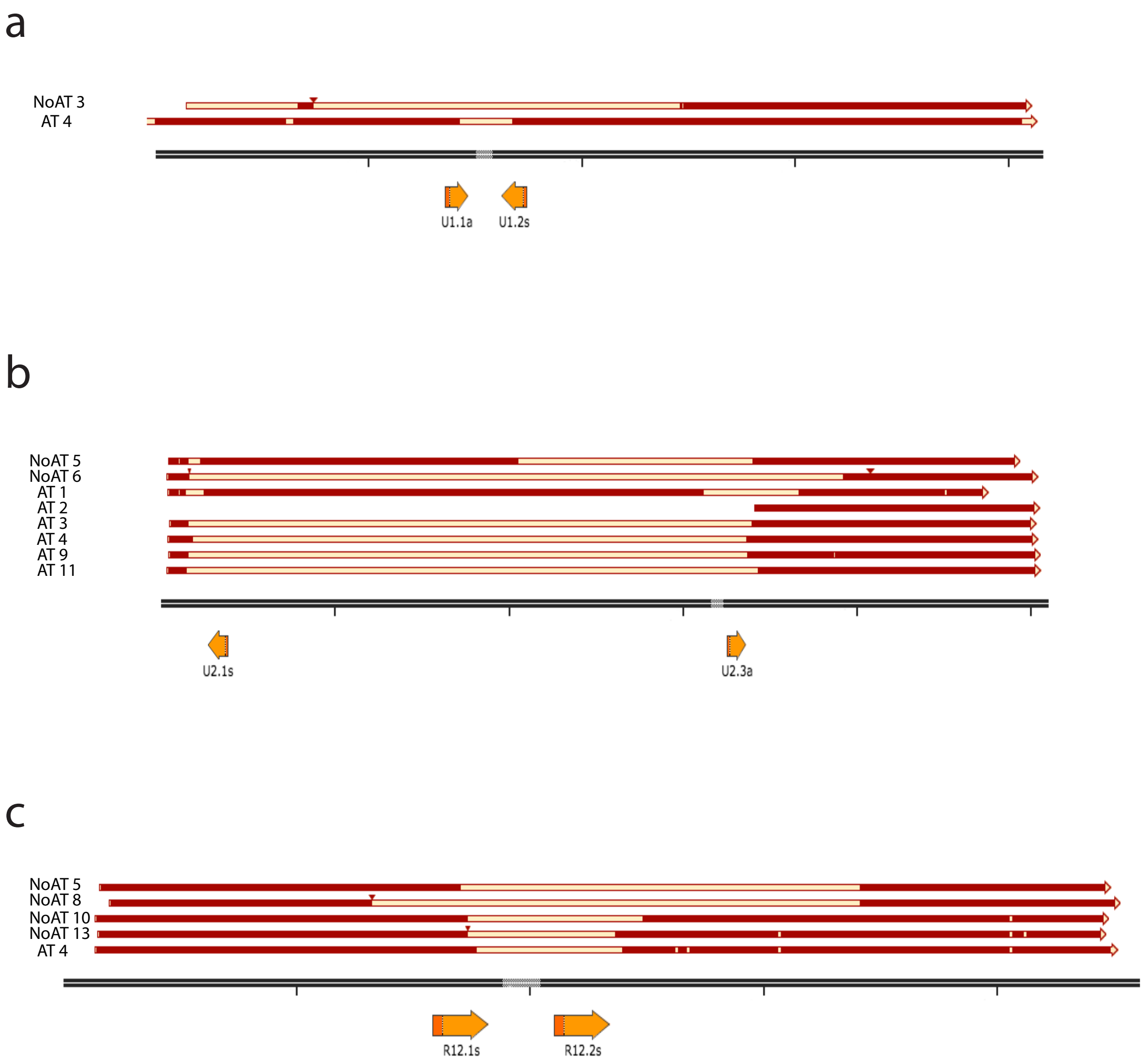

Suppl. Fig. 3 Agarose gel electrophoresis of *Ubn1* deletion, 5' and 3' flanking region wildtype PCR products from biopsies of mice obtained from C57BL/6J zygote electroporation of *Ubn1* Cas12a RNP complexes. Molecular weight marker (M), positive control DNA from ESCs carrying a *Ubn1* deletion (+), negative control wild type DNA (WT), no template control (-), and 8 samples obtained from mice (#1 to #8) are shown. The genotype of each mouse is given (below); alleles are categorized for deletion (Del) and wild type (WT). The positions of the 500 bp (black arrow head) and 1 kb (white arrow head) size marker fragments are indicated.

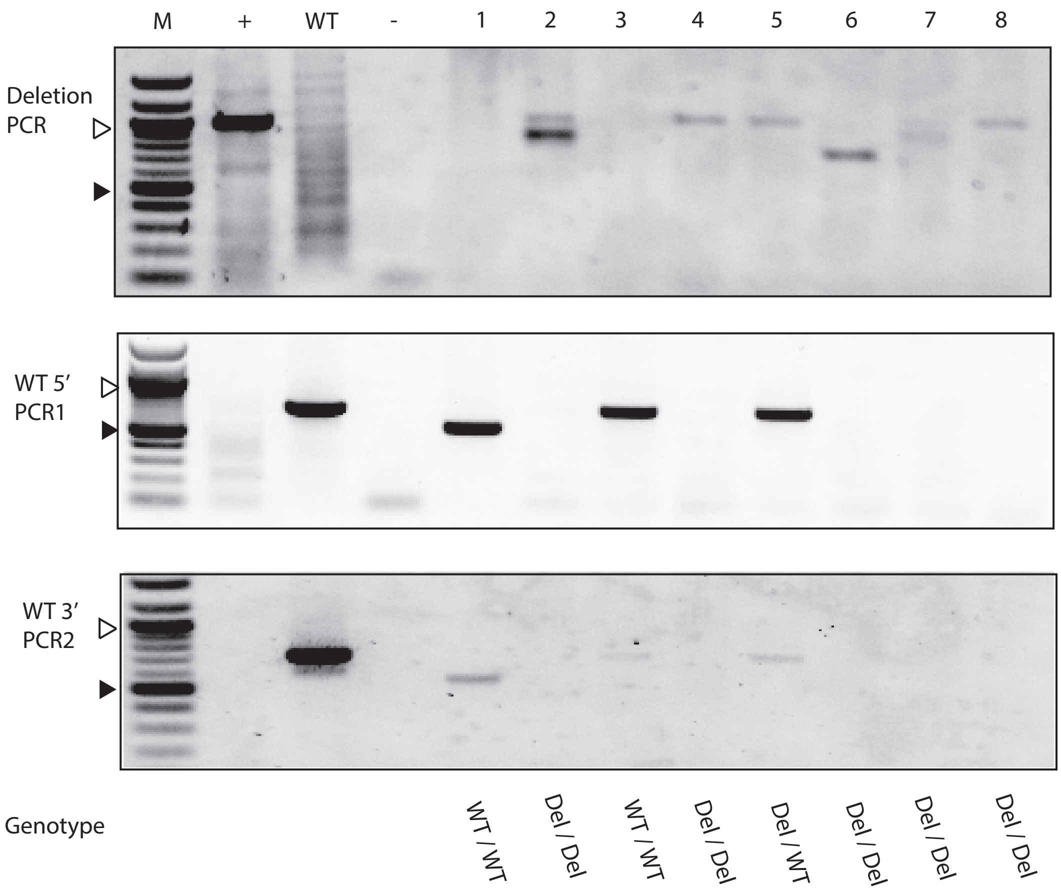

Supplement: Supplementary file 1 — Supplementary file1 (PDF 340 kb) [file 11248_2019_168_MOESM1_ESM.pdf]
